# Mg-Doped P-Type AlN Thin Film Prepared by Magnetron Sputtering Using Mg-Al Alloy Targets

**DOI:** 10.3390/mi16091035

**Published:** 2025-09-10

**Authors:** Yulin Ma, Xu Wang, Kui Ma

**Affiliations:** 1 Department of Electronic Science, Guizhou University, Guiyang 550025, China; mayulin@zuaa.zju.edu.cn (Y.M.);; 2 Key Laboratory of Micro-Nano-Electronics and Software Technology of Guizhou Province, Guiyang 550025, China; 3 Semiconductor Power Device Reliability Engineering Research Center of Ministry of Education, Guiyang 550025, China

**Keywords:** Aluminum nitride, Mg-doping, p-type, magnetron sputtering, high-temperature annealing

## Abstract

Aluminum nitride (AlN), a III-V wide-bandgap semiconductor, has attracted significant attention for high-temperature and high-power applications. However, achieving p-type doping in AlN remains challenging. In this study, p-type AlN thin films were fabricated via magnetron sputtering using Mg-Al alloy targets with varying Mg concentrations (0.01 at.%, 0.02 at.%, and 0.5 at.%), followed by ex situ high-temperature annealing to facilitate Mg diffusion and electrical activation. The structural, morphological, and electrical properties of the films were systematically characterized using X-ray diffraction (XRD), white light interferometry (WLI), scanning electron microscopy (SEM), energy-dispersive X-ray spectroscopy (EDS), X-ray photoelectron spectroscopy (XPS), and Hall effect measurements. The results demonstrate that at a Mg doping concentration of 0.02 at.%, the films exhibit optimal crystallinity, uniform Mg distribution, and a favorable balance between carrier concentration and mobility, resulting in effective p-type conductivity. Increasing Mg doping leads to higher surface roughness and the formation of columnar and conical grain structures. While high Mg doping (0.5 at.%) significantly increases carrier concentration and decreases resistivity, it also reduces mobility due to enhanced impurity and carrier–carrier scattering, negatively impacting hole transport. XPS and EDS analyses confirm Mg incorporation and the formation of Mg-N and Al-Mg bonds. Overall, this study indicates that controlled Mg doping combined with high-temperature annealing can achieve p-type AlN films to a certain extent, though mobility and carrier activation remain limited, providing guidance for the development of high-performance AlN-based bipolar devices.

## 1. Introduction

As one of the representatives of fourth-generation semiconductors, Aluminum nitride (AlN) exhibits significant advantages in high-temperature and high-power applications, attributed to its ultra-wide bandgap of 6.2 eV, high breakdown electric field at 12 MV·cm^−1^, and exceptional thermal conductivity of 3.4 W·cm^−1^·K^−1^ [1,2,3,4,5,6,7]. Presently, substantial research centers on the physical properties of AlN, notably p-type doping, recognized as crucial for advancing AlN-based bipolar devices [8,9,10,11,12]. The efficacy of hole injection into the active region and electron leakage control hinges on the quality of p-type doping [13]. Nevertheless, p-type doping in AlN presents a particular challenge relative to n-type [14,15], with beryllium (Be), magnesium (Mg), and zinc (Zn) serving as common p-type dopants in III-nitrides [16]. Sequentially increasing activation energies are characteristic for these elements in AlN [17]. Although Be exhibits a lower activation energy relative to other elements, its toxicity and tendency to introduce interstitial atoms that offset acceptors render it less favorable. Consequently, Mg is the prevalent acceptor dopant for p-type doping purposes [18]. The revelation of hydrogen passivation for Mg acceptors and thermal annealing’s role in activating Mg within GaN has markedly resolved p-type doping issues in GaN [19,20]. Nonetheless, Mg doping in AlN encounters substantial difficulties [21].

To address the challenges of p-type doping in AlN, this study designed Mg-Al alloy targets with varying Mg concentrations (0.01 at.%, 0.02 at.%, 0.5 at.%) and deposited AlN: Mg films via RF sputtering in a nitrogen-rich ambiance. To augment the doping efficiency of Mg and mitigate the self-compensation phenomenon, an ex situ high-temperature annealing procedure was employed to facilitate Mg diffusion and ensure electrical activation within AlN, thereby realizing effective p-type doping.

## 2. Materials and Methods

**Scheme 1:**
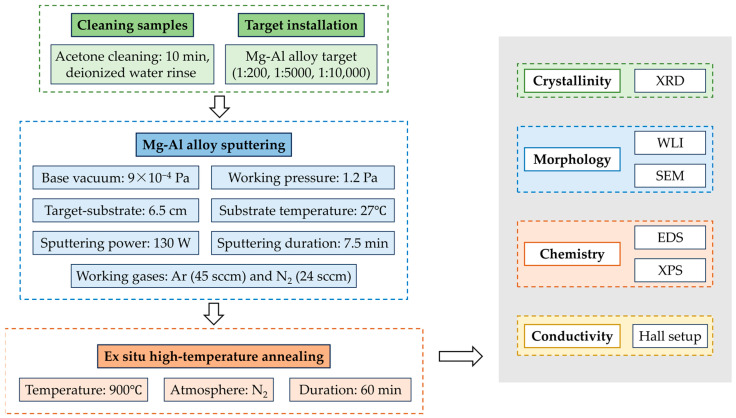
Technical roadmap for the preparation of p-type AlN: Mg thin films by magnetron sputtering using Mg-Al alloy targets.

**Figure 1:**
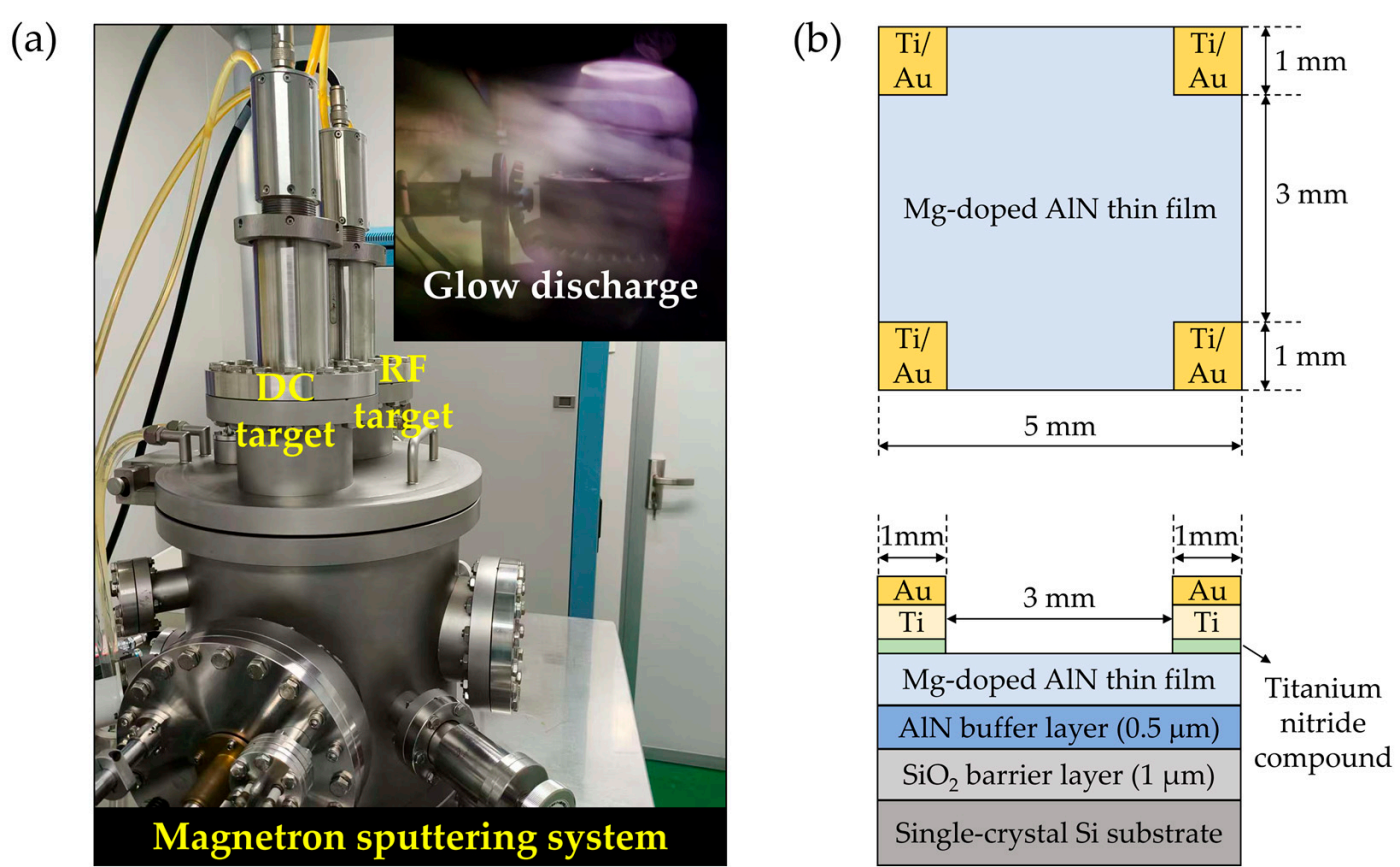
(**a**) Photograph of the magnetron sputtering system. The DC target is located on the left, and the RF target on the right, positioned side by side. An automatic transfer mechanism enables lateral sample movement between the two targets. The inset in the upper right shows a glow discharge during sputtering of the Mg–Al alloy target. (**b**) Schematic top and front views of the sample after Ti/Au electrode deposition by sputtering.

As depicted in Figure 1a, the initial step involved cleaning Si/SiO_2_ (1 μm)/AlN (0.5 μm), provided by an external collaborator, through ultrasonic agitation in acetone for 10 min to eliminate surface contaminants. Following this, the substrates underwent triple rinsing with deionized water to eradicate residual acetone. The samples were then carefully placed face-up at the center of the base using antistatic tweezers. Subsequently, Mg-doped AlN films were fabricated using a high-vacuum dual-target magnetron sputtering setup (JGP280, SKY Technology Development Co., Ltd., Chinese Academy of Sciences, Shenyang, China) powered by RF source (PG-500, Gmpower Technology Co., Ltd., Beijing, China), as shown in Figure 1a. The power stability of the RF source is ≤±0.5%, with an output frequency of 13.56 MHz and a standard sine wave frequency signal. The matching system was set to manual mode, and by repeatedly pressing the Ca+ button on the panel, the reflected power was adjusted to 0 W. The targets consisted of Mg-Al alloys with Mg concentrations of 0.01 at.%, 0.02 at.%, and 0.5 at.%, respectively. The purity of the targets was 99.99%, with a diameter of 60 mm and a thickness of 5 mm (Equipment from Shijiazhuang Dongming New Materials Technology Co., Ltd., Shijiazhuang, China). When sputtering started, the targets emitted a bright blue-white glow, primarily due to strong aluminum emission lines concentrated in the blue and green regions (e.g., 396.15 nm and 394.40 nm, both purple-blue, as well as several green lines). These lines are particularly intense, while the strongest magnesium emission line at 285.2 nm is in the ultraviolet range and is invisible to the naked eye. The argon plasma itself also emits a strong blue-violet light. Therefore, the glow from the Mg-Al alloy targets typically appears bright with a blue-violet base and a distinct greenish hue, overall exhibiting a blue-white color, as shown in Figure 1a. Comprehensive sputtering parameters are detailed in Scheme 1. Post-deposition, samples were heat-treated in a horizontal tube furnaceTwo-tube design, Qingdao Xuguang Instrument Equipment Co., Ltd., Qingdao, China) under nitrogen at 900 °C for one hour. After natural cooling to room temperature, the samples were examined using an optical microscope, and the AlN film surfaces were found to remain intact without any cracks.

The crystal structure of the samples was characterized using a X-ray diffraction instrument(XRD; SmartLab9, Rigaku Corporation, Tokyo, Japan). Surface and cross-sectional morphologies were analyzed with a white light interferometer (WLI; Bruker Contour GT-K 3D, Bruker Corporation, Karlsruhe, Germany) and a scanning electron microscope (SEM; Hitachi Regulus 8100, Hitachi High-Technologies Corporation, Tokyo, Japan). The atomic percentage of Mg and the chemical composition within the AlN films were determined by energy dispersive X-ray spectroscopy (EDS; equipped with the SEM) and an X-ray photoelectron spectrometer (XPS; Thermo Fisher K-Alpha High-Performance Compact, Thermo Fisher Scientific, Waltham, MA, USA). Resistivity, carrier concentration, and Hall mobility on the sample surfaces were measured employing a Hall effect measurement system (VDP6800, SmartTech, Taiwan, China).

During Hall effect measurements on the films, we found that the absence of electrodes led to unsatisfactory results, mainly because the contact points failed to form proper ohmic contacts with the films. To ensure accurate measurement, ohmic contact electrodes were fabricated prior to Hall testing. Since the effectiveness of ohmic contact formation varies with electrode metals, previous studies have reported that Ti/Au or Ni/Au structures are typically required to achieve reliable ohmic contacts on p-type AlN films. To fabricate ohmic contact electrodes at four designated positions on the film surface, the following process was employed. A spin coater (KW-5, Institute of Microelectronics, Chinese Academy of Sciences, Beijing, China) was first used to uniformly coat the sample surface with negative photoresist AZ_NLOF 2035 at 3000 rpm for 1 s. After spin coating, the sample was left to rest for 2 min, followed by prebaking at 110 °C for 2 min. Lithography was then carried out using a laser direct-writing lithography system (MLA100, Heidelberg Instruments GmbH, Heidelberg, Germany) with an i-Line light source, an exposure dose of 2100 mJ/cm^2^, and a focus value of 0. Post-exposure baking was conducted at 110 °C for 2 min, followed by another 2 min rest. The sample was subsequently developed in ZX-238 developer for 2 min to form four electrode windows. After development, the sample was rinsed with deionized water and dried with nitrogen gas for subsequent processing. The patterned sample with four electrode windows was then placed into a magnetron sputtering system, where Ti (99.99% purity, 60 mm diameter, 5 mm thickness, Shijiazhuang Dongming New Materials, Shijiazhuang, China) and Au (99.99% purity, 60 mm diameter, 0.5 mm thickness, Shijiazhuang Dongming New Materials) layers were sequentially deposited. After sputtering, the photoresist was removed using acetone, followed by deionized water rinsing to clean the sample surface. Finally, the sample was subjected to rapid thermal annealing at 400 °C for 1 min in a high-temperature diffusion furnace under nitrogen ambient, in order to improve the ohmic contact between the electrodes and the film. The detailed sputtering parameters are summarized in Table 1, and a schematic diagram of the fabricated electrode structure is shown in Figure 1b.

## 3. Results

### 3.1. XRD

Although multiple thin films (total thickness ~1.5 μm or more) were deposited on the silicon substrate, the penetration depth of X-rays is much greater than this thickness. As a result, the incident X-rays can easily pass through the films and reach the underlying silicon substrate, producing sharp and intense substrate diffraction peaks in the XRD patterns. This is a common phenomenon in heteroepitaxial film XRD measurements. To minimize the interference of substrate peaks on the analysis of Mg-doped AlN epilayers, adjustments to the X-ray incident angle were made during the measurements.

**Figure 2:**
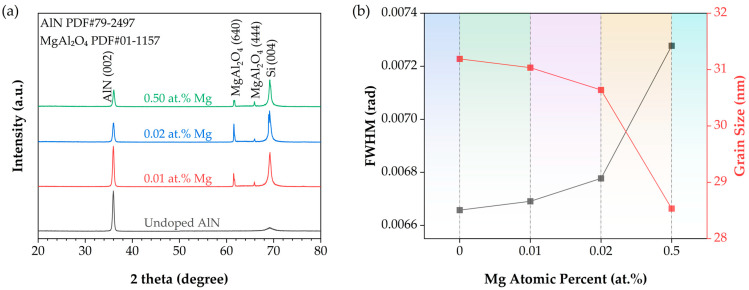
(**a**) XRD patterns of samples with different Mg doping concentrations: undoped (black), 0.01 at.% Mg (red), 0.02 at.% Mg (blue), and 0.50 at.% Mg (green).; (**b**) Line plot showing the variation in XRD FWHM (black, obtained from 2θ scans) and grain size (red) with Mg doping concentration.

XRD measurements were carried out on undoped AlN films as well as Mg-doped AlN films with three different doping concentrations (0.01 at.%, 0.02 at.%, and 0.5 at.%). The results are shown in Figure 2. For the undoped sample, two distinct diffraction peaks were observed: the main peak at ~35.96° corresponds to the AlN (002) reflection, confirmed by comparison with the AlN reference card (PDF#79-2497), while the secondary peak at ~69.12° originates from the silicon substrate and was identified as the Si (004) reflection (PDF#27-1402). For the Mg-doped films, all samples exhibited the AlN (002) peak near 35.96°, confirming the hexagonal wurtzite structure (α-AlN) [22]. Notably, with increasing Mg concentration, the AlN (002) peak gradually shifted toward lower angles. This behavior is consistent with previous reports on Cr- and Ge-doped AlN films [23,24,25], indicating that interstitial atoms and defects cause lattice expansion along the c-axis, resulting in the observed peak shift. When Mg atoms substitute Al sites in the lattice, expansion occurs, especially at higher doping levels, where increased stress between Mg and the AlN lattice disrupts crystal order and reduces crystallinity. The expansion effect becomes more pronounced with increasing concentration, reaching a maximum at 0.5 at.%. Excessive doping leads to a degradation in film crystallinity, as evidenced by the reduced diffraction peak intensity. High Mg concentrations may also introduce lattice defects or dislocations, further impeding crystallization. Consequently, the sample with a doping concentration of 0.01 at.% exhibited relatively high crystallinity, with its diffraction peak intensity second only to the undoped sample; whereas the 0.5 at.% sample, due to its relatively high doping level, exhibited comparatively poorer crystallinity.

In addition, two extra peaks appeared at ~61.40° and 65.84° in Mg-doped AlN samples with different concentrations. Considering the sample structure (Si/SiO_2_/AlN/Mg-doped AlN) and the Cu Kα radiation source, these peaks are most likely derived from the same secondary phase. Comparison with the MgAl_2_O_4_ spinel reference card (PDF#01-1157) identified them as the MgAl_2_O_4_ (640) and (444) reflections, respectively. The formation of MgAl_2_O_4_ can be attributed to the presence of oxygen. Specifically, the SiO_2_ interlayer in the sample structure may decompose under the high temperatures required for subsequent AlN and Mg-doped AlN growth, releasing free oxygen. The released oxygen can diffuse upward and react with Al and N from AlN, as well as Al, N, and Mg from Mg-doped AlN, leading to the formation of MgAl_2_O_4_ spinel.

The grain size was calculated using the Debye–Scherrer equation, as follows:

D = Kλ/(βcos θ),(1)

where D is the grain size (nm), K is the Scherrer constant (0.89), λ is the X-ray wavelength (0.15406 nm for Cu K_α_ radiation), β is the full width at half maximum (FWHM, converted to radians), and θ is the diffraction angle (also in radians). Figure 2b shows the variation in FWHM and grain size as a function of Mg doping concentration. With increasing Mg concentration, the FWHM of the (002) peak decreases while the grain size increases. These results indicate that higher Mg doping concentrations deteriorate the crystallinity of the films under the present growth conditions.

### 3.2. WLI

**Figure 3:**
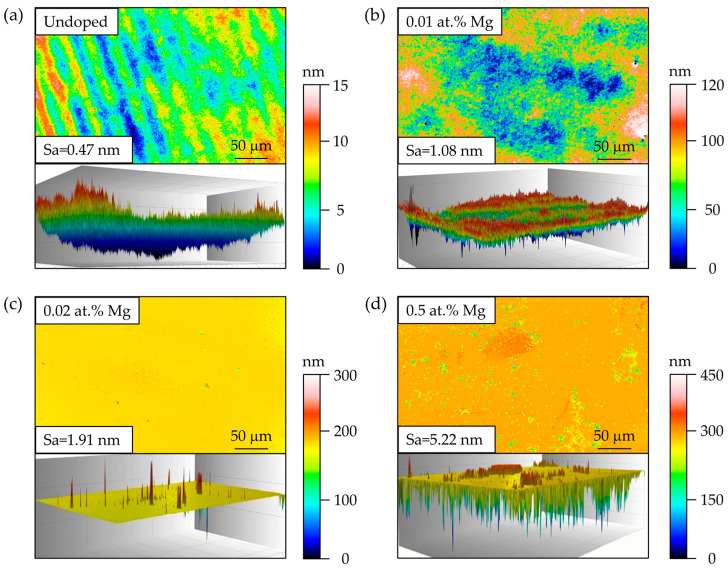
Surface morphology of AlN films with different Mg doping concentrations: (**a**) undoped, (**b**) 0.01 at.% Mg, (**c**) 0.02 at.% Mg, and (**d**) 0.50 at.% Mg. Corresponding 3D images are shown for each sample.

As shown in Figure 3, WLI analysis reveals that the undoped AlN film exhibits a relatively smooth surface within the 20 nm scale, characterized by a typical step-flow growth mode. With increasing Mg doping concentration, the surface roughness gradually increases. Conical structures become evident in the 0.02 at.% Mg-doped sample, while in the 0.5 at.% Mg-doped film, three-dimensional imaging clearly displays numerous conical and irregular block-like (surface cluster) features. The conical protrusions are generally associated with columnar grain growth and the geometric “shadowing” effect: higher Mg incorporation reduces the diffusion length of surface adatoms and increases the nucleation density, enabling grains at step edges or defect sites to grow preferentially. The continuous extension of such grains along the vertical direction results in tapered, cone-like morphologies. In contrast, the irregular block-like clusters are mainly related to localized compositional enrichment and nonuniform stress distribution. Mg segregation at the surface or grain boundaries, together with point defects introduced by substitutional Mg, modifies surface energy and adatom mobility, thereby promoting defect-mediated island coalescence and surface roughening. This drives the film toward a Volmer–Weber island growth mode, ultimately forming clusters of varied size and shape. At higher doping levels, enhanced Mg diffusion and an incipient tendency toward phase separation can produce Mg-rich regions at grain boundaries or on the surface, which act as heterogeneous nucleation sites and evolve into clusters. Meanwhile, the increased and uneven lattice stress directs crystal growth preferentially along certain orientations, further facilitating the development of columnar/conical structures and markedly intensifying surface roughening.

### 3.3. SEM and EDS

**Figure 4:**
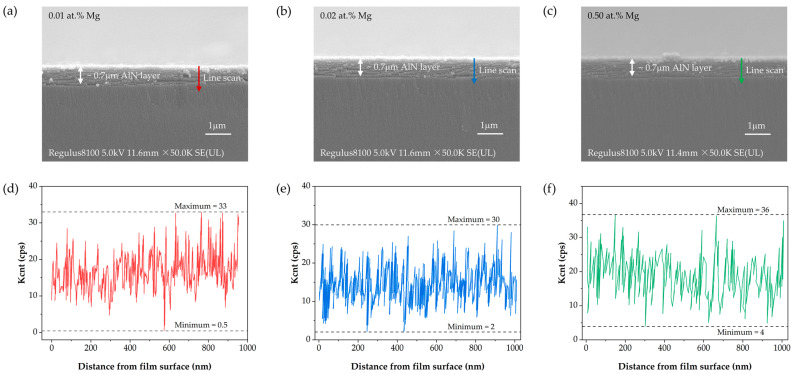
Cross-sectional SEM images and corresponding EDS energy scans of AlN films with different Mg doping concentrations: (**a**) 0.01 at.% Mg SEM, (**b**) 0.02 at.% Mg SEM, (**c**) 0.50 at.% Mg SEM, (**d**) 0.01 at.% Mg EDS, (**e**) 0.02 at.% Mg EDS, (**f**) 0.50 at.% Mg EDS. As the Mg concentrations are below the EDS quantitative limit, the Mg signals are shown for qualitative reference only.

As shown in Figure 4, SEM and EDS analyses were performed on three AlN films with different Mg doping concentrations to further examine the content and distribution of Mg within the layers. Figure 4a–c present the cross-sectional SEM images of the 0.01 at.% Mg, 0.02 at.% Mg, and 0.5 at.% Mg samples, respectively. It can be observed that, due to the relatively low overall Mg doping concentrations, the samples exhibit similar morphology in terms of cross-sectional features and film thickness at the micrometer scale. Since AlN films exhibit poor electrical conductivity, the samples were sputter-coated with a thin Au layer for ~5 min prior to measurement. As a result, a small number of Au nanoparticles with a size of approximately 10 nm are occasionally visible on the surface; however, their influence on the subsequent analysis is negligible. From the scale bars, the thickness of the AlN layers is estimated to be ~0.7 μm, including a ~0.5 μm AlN buffer layer. The scan trajectories along the film depth direction are also indicated. Figure 4d–f display the corresponding EDS energy scan results. Because the Mg doping concentrations (0.01 at.%, 0.02 at.%, and 0.5 at.%) are all below the quantitative detection limit of the employed EDS system (approximately 1 at.%), the Mg signals appear extremely weak and do not provide reliable quantitative distribution information. This demonstrates that EDS is not suitable for effectively characterizing the Mg dopant distribution in this study. Therefore, XPS was subsequently employed to analyze the near-surface region of the films (<10 nm) with higher precision.

### 3.4. XPS

**Figure 5:**
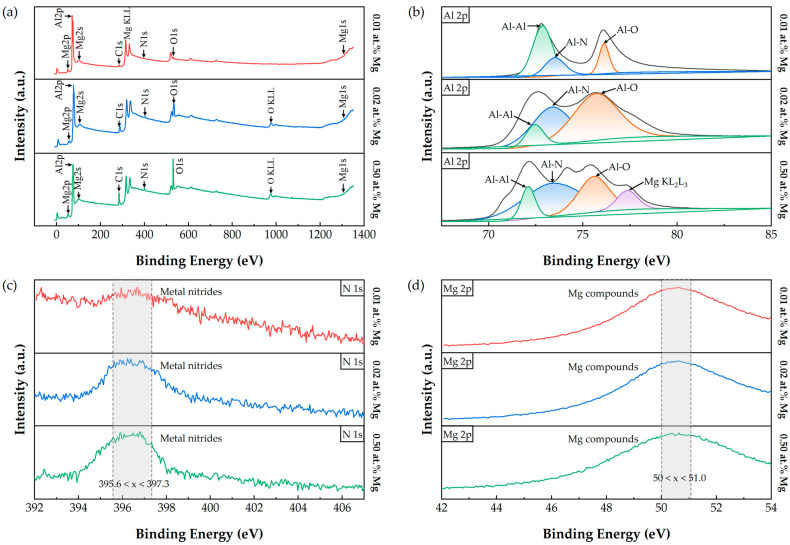
XPS spectra of AlN films with varying Mg doping concentrations: (**a**) survey spectra, (**b**) Al 2p high-resolution spectra, (**c**) N 1s high-resolution spectra, and (**d**) Mg 2p high-resolution spectra. The spectra illustrate the chemical states at different doping levels and the incorporation of Mg in the films.

We then performed XPS measurements to investigate the chemical bonding states in the samples, acquiring survey spectra as well as high-resolution C 1s, Al 2p, N 1s, and Mg 2p regions; the results are presented in Figure 5. Because the samples are non-carbon-based, all spectra were charge-referenced to the adventitious carbon C–C/C–H peak at 284.8 eV. The survey spectrum in Figure 5a confirms the presence of C, O, Al, N, and Mg. Figure 5b shows the high-resolution Al 2p spectra, where Al–Al (~72.5 eV), Al–N (~73.6 eV), and Al–O (~74.5 eV) chemical states can be identified. The feature near ~77.48 eV may be influenced by Mg-related Auger peaks or energy overlap. Under certain measurement conditions, metallic Al can exhibit spin–orbit splitting or asymmetric line shapes (as seen for Al–Al); for simplicity, we did not perform detailed deconvolution of the Al–Al component, and spin-split components were not explicitly separated for other Al chemical states. Figure 5c presents the N 1s spectra, showing a single peak at ~396.36 eV, consistent with N–metal bonding [26]. Figure 5d displays the Mg 2p region, where the peak at ~50.2 eV indicates that Mg is present in a chemical (compound) state rather than as metallic Mg. It should be noted that XPS alone cannot unambiguously determine whether Mg has substituted into Al lattice sites in AlN; therefore, Hall measurements were performed subsequently to evaluate the electrical effects of Mg incorporation.

### 3.5. Hall Effect Measurement

As shown in Table 2, Hall measurements confirm that Mg doping successfully converts AlN films from n-type to p-type conductivity; however, the overall doping effectiveness remains limited. It should be specifically noted that all Hall measurements for Mg-doped films in this study were conducted after annealing. It should be noted that the relatively low resistivity of undoped AlN may result from the presence of p-type or n-type impurities. Hall measurements directly indicate the conduction type, which provides indirect insight into the elements contributing to the conductivity. The undoped AlN film exhibits n-type behavior, with a carrier concentration of 1.24 × 10^15^ cm^−3^ and a resistivity of approximately 1014.72 Ω·cm. After Mg incorporation, all films display p-type conduction, indicating that Mg atoms effectively introduce acceptor levels. Nevertheless, the electrical parameters reveal that the practical effectiveness of p-type doping is rather unsatisfactory. At 0.01 at.% Mg, resistivity decreases markedly to 644.12 Ω·cm, but the Hall mobility drops drastically to 0.105 cm^2^·V^−1^·s^−1^, suggesting that impurity and defect scattering strongly suppress carrier transport. When the Mg concentration increases to 0.02 at.%, carrier concentration rises slightly to 4.77 × 10^15^ cm^−3^, resistivity further decreases to 254.59 Ω·cm, and mobility recovers to 0.737 cm^2^·V^−1^·s^−1^, indicating a temporary optimization. However, at higher doping levels (0.50 at.% Mg), although the carrier concentration significantly increases to 9.94 × 10^16^ cm^−3^ and resistivity is further reduced to 49.03 Ω·cm, mobility decreases again to 0.181 cm^2^·V^−1^·s^−1^. This behavior reflects the strong impurity scattering and carrier–carrier interactions induced by excessive doping, which hinder effective hole transport. It is worth noting that the experimental results for resistivity, mobility, and carrier concentration are not fully self-consistent, since calculations based on the theoretical relation

ρ = 1/(q·n·μ),(2)

do not perfectly match the tabulated data. Possible reasons include measurement uncertainties in Hall testing, particularly for highly resistive samples; High defect density in AlN films, which may create deep trap states that capture carriers and reduce their contribution to conduction; Limited activation efficiency of Mg acceptors, resulting in discrepancies between nominal and effective dopant concentrations; And surface charge accumulation or contact resistance effects during measurements, further amplifying deviations. Overall, although p-type doping was achieved in this work, the relatively low mobility, limited carrier activation efficiency, and measurement uncertainties indicate that Mg doping in AlN still faces inherent challenges in realizing efficient p-type conductivity.

## 4. Discussion

This study demonstrates that Mg doping significantly influences the structural, morphological, and electrical properties of AlN films deposited by magnetron sputtering using Mg-Al alloy targets. XRD analysis indicates that at low doping concentrations (0.01–0.02 at.% Mg), the films retain the hexagonal wurtzite structure (α-AlN), with sharp and intense (002) diffraction peaks reflecting good crystallinity. As the doping concentration increases to 0.5 at.% Mg, the (002) peak slightly shifts to lower angles, broadens, and decreases in intensity, suggesting lattice expansion and stress accumulation that degrade crystallinity. This is accompanied by increased surface roughness and the formation of columnar grain structures, as observed in WLI and SEM 3D images. Surface morphology analysis reveals conical protrusions and irregular clustered structures at high doping levels, attributed to lattice stress, local compositional enrichment, and increased point defects caused by Mg incorporation.

EDS and XPS analyses confirm that Mg is successfully incorporated into the AlN lattice, forming Mg–N and Al–Mg bonds. However, the uniformity of Mg distribution is limited, particularly at low doping levels where EDS signals are too weak for reliable quantification. Hall measurements show that undoped AlN is n-type with very high resistivity and low carrier concentration. After Mg doping, films become p-type, with carrier concentration increasing with doping level, while Hall mobility remains generally low. At low doping levels, mobility decreases to the order of 0.1 cm^2^·(V·s)^−1^, indicating strong impurity and defect scattering that restricts hole transport. At high doping (0.50 at.% Mg), despite the significant increase in carrier concentration, mobility decreases again, reflecting enhanced impurity scattering and carrier–carrier interactions that limit effective transport. Overall, p-type doping of AlN is achieved, but low mobility and limited activation efficiency highlight the inherent challenges in realizing highly efficient p-type AlN. The optimal compromise occurs at 0.02 at.% Mg, where the film exhibits relatively good crystallinity, reduced resistivity, and moderate carrier concentration.

## 5. Conclusions

In this work, p-type AlN films with different Mg doping concentrations (0.01 at.%, 0.02 at.%, and 0.5 at.%) were successfully prepared using Mg-Al alloy targets, and their structural, surface morphological, and electrical properties were systematically investigated. The results show that at low doping levels, the films retain the hexagonal wurtzite structure, with sharp and intense (002) diffraction peaks indicating good crystallinity, whereas at a high doping level of 0.5 at.% Mg, the peak shifts slightly to lower angles, broadens, and decreases in intensity, reflecting lattice expansion and stress accumulation that degrade crystallinity. Surface analysis reveals increased roughness in highly doped films, accompanied by the formation of columnar, conical, and irregular clustered structures, attributed to lattice stress, local compositional enrichment, and increased point defects. XPS and EDS confirm successful incorporation of Mg into the AlN lattice, forming Mg–N and Al–Mg bonds, although quantitative analysis by EDS is limited at low doping levels. Notably, a certain amount of oxygen remains in the films, mainly originating from the decomposition of the SiO_2_ interlayer during high-temperature annealing; some of this oxygen reacts with Al and Mg to form MgAl_2_O_4_ spinel, which may influence local structure and electrical properties. Hall measurements indicate that Mg doping successfully converts the films from n-type to p-type, but mobility remains low; at low doping levels, carrier transport is limited by impurity and defect scattering, while at high doping, despite significantly increased carrier concentration, mobility decreases again due to strong impurity scattering and carrier–carrier interactions. Overall, the 0.02 at.% Mg-doped film achieves the best compromise among crystallinity, electrical performance, and uniform Mg distribution, exhibiting optimal overall performance. This study demonstrates that Mg-Al alloy targets combined with post-deposition annealing can realize p-type doping in AlN, providing a feasible approach for further performance optimization while also highlighting the intrinsic challenges in achieving highly efficient p-type AlN.

## Figures and Tables

**Table 1:** DC sputtering parameters for Ti/Au electrodes.

Target	Base Vacuum (Pa)	Working Pressure (Pa)	Target-to-Substrate Distance (cm)	Substrate Temperature (°C)	DC Power (W)	Time (min)	Ar Flow Rate (sccm)
Ti	9.0 × 10^−4^	0.8	6.5	27	60	3	25
Au	9.0 × 10^−4^	0.5	6.5	27	24	15	25

**Table 2:** Hall measurement results of AlN films with varying Mg doping concentrations, including carrier type, Hall mobility, carrier concentration, and resistivity.

Sample	Type	Hall Mobility (cm^2^·V^−1^·s^−1^)	Carrier Concentration (cm^−3^)	Resistivity (Ω·cm)
Undoped AlN	n	4.46	1.24 × 10^15^	1014.72
0.01 at.% Mg	p	0.105	3.39 × 10^15^	644.12
0.02 at.% Mg	p	0.737	4.77 × 10^15^	254.59
0.50 at.% Mg	p	0.181	9.94 × 10^16^	49.03

## Data Availability

The raw/processed data required to reproduce these findings cannot be shared at this time as the data also forms part of an ongoing study.
